# Morphological and Chemical Study of Pathological Deposits in Human Aortic and Mitral Valve Stenosis: A Biomineralogical Contribution

**DOI:** 10.1155/2015/342984

**Published:** 2015-01-19

**Authors:** Valentina Cottignoli, Elena Cavarretta, Loris Salvador, Carlo Valfré, Adriana Maras

**Affiliations:** ^1^Department of Earth Sciences, Sapienza University of Rome, Piazzale Aldo Moro 5, 00185 Rome, Italy; ^2^Department of Medical-Surgical Sciences and Biotechnologies, Sapienza University of Rome, Corso della Repubblica 79, 04100 Latina, Italy; ^3^Division of Cardiac Surgery, San Bortolo Hospital, Viale Rodolfi 37, 36100 Vicenza, Italy

## Abstract

Aim of this study was to investigate heart valve calcification process by different biomineralogical techniques to provide morphological and chemical features of the ectopic deposit extracted from patients with severe mitral and aortic valve stenosis, to better evaluate this pathological process. Polarized light microscopy and scanning electron microscopy analyses brought to light the presence of nodular and massive mineralization forms characterized by different levels of calcification, as well as the presence of submicrometric calcified globular cluster, micrometric cavities containing disorganized tissue structures, and submillimeter pockets formed by organic fibers very similar to amyloid formations. Electron microprobe analyses showed variable concentrations of Ca and P within each deposit and the highest content of Ca and P within calcified tricuspid aortic valves, while powder X-ray diffraction analyses indicated in the nanometer range the dimension of the pathological bioapatite crystals. These findings indicated the presence of highly heterogeneous deposits within heart valve tissues and suggested a progressive maturation process with continuous changes in the composition of the valvular tissue, similar to the multistep formation process of bone tissue. Moreover the micrometric cavities represent structural stages of the valve tissue that immediately precedes the formation of heavily mineralized deposits such as bone-like nodules.

## 1. Introduction

Several physiological and pathological mineralized deposits are present in the human body. The major mineralized products (enamel, dentin, and bone) are formed by well-controlled processes of mineralization [1, 2]. Other deposits (e.g., dental calculus, urinary stones, arthritic cartilage, and heart valve calcification) are produced by pathologic mineralization. Calcific aortic valve stenosis is a major health problem facing our aging society and, after hypertension and coronary artery disease, is the third most common cardiovascular disorder and the most common indication for surgical valve replacement in the United States [3]. Despite the fact that ectopic deposits within heart valve tissues are intensively investigated, their formation mechanism is still unclear, and currently there are no effective medical treatments to delay valve calcification course progression.

The calcified deposits are composed of mineral and organic components and represent inorganic phases produced by the activity of living organisms, better known as biominerals [4]. Most pathologic biomineral deposits, generically termed calcifications or ectopic calcifications, are composed of a mixture of calcium phosphate phases. The formation of each phase depends on the combination of pH, temperature, and solution composition [5–8], and the characteristics of these deposits are different from normal mineralized tissues and from their inorganically produced counterparts. As the formation of biominerals is associated with different processes involving complex physicochemical and biological mechanisms, we sought to better comprehend this pathological process by conducting an interdisciplinary study focusing on the biomineralogical features of the ectopic deposits in heart valves and to present a novel biomineralogical approach that may provide important insights into the growth mechanisms of pathological events.

## 2. Materials and Methods

### 2.1. Valve Samples

Calcified aortic valves (tricuspid type, *n* = 29; bicuspid type, *n* = 3) and mitral valves (*n* = 4) were obtained from Caucasian patients of both sexes and different ages (41–90 years old) (Table 1). Samples were collected as resection materials from patients undergoing valve replacement due to severe aortic valve and mitral stenosis. All surgical interventions were performed at the Department of Cardiac Surgery, Treviso Regional Hospital, Italy. An institutional committee approved the study, and the patients gave written informed consent. Calcified valves without any sign of inflammation were included in this study. For a mineralogical preparation of the samples, after extraction tissues were preserved and dehydrated in absolute alcohol, then to sterilize the biological material, samples were exposed to UV radiation for almost 72 hours.

### 2.2. Preparation of Polished Thin Sections

**Figure 1:**
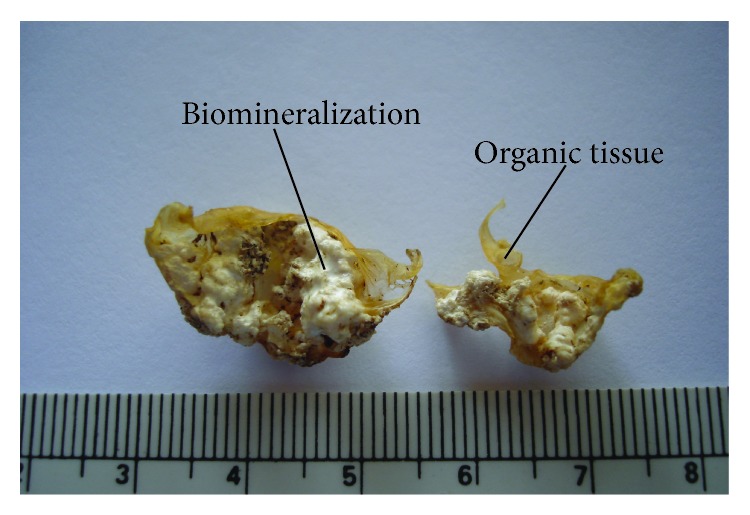
Macroscopic images of a large mineralized deposit on a valve leaflet after the mineralogical preparation (dehydration in absolute alcohol and exposure to UV radiation) of the sample. The white deposit represents the biomineral formation grown within the organic tissue (in yellow) of the human heart valve.

As the dehydration treatment of the samples gave to the calcified valves the consistency of a soft rock (Figure 1) the same procedure used for rock samples was applied for the preparation of polished thin sections (PTS) of calcified deposits. Only samples with an extensive calcification were chosen for PTS, because the preparation method requires a significant amount of calcification to obtain good-quality PTS. The epoxy mounting method was used to prepare PTS with 30 *μ*m thicknesses [9, 10].

### 2.3. Polarized Light Microscopy and Electron Microprobe Analyses

PTS were studied with an Olympus BX60 microscope with transmitted- and reflected-polarized light (PLM). Cross polarized light was used to test birefringence of samples. PTS were then coated with a thin layer of graphite, sputter-coated with an Edwards E306A High Vacuum System, and analyzed with a Cameca SX 50 EMP equipped with four wavelength dispersive spectrometers (WDS) and one energy dispersive spectrometer (EDS), at a beam current of 15.0 nA and an accelerating voltage of 15 kV. The samples were investigated using a defocused beam, as described by Ballirano et al. [11]. Standards used for the quantitative chemical analyses were all synthetic, apart from the natural fluorapatite from Durango (Mexico) used for phosphorus (P). The synthetic standards used were barite for sulfur (S), periclase for magnesium (Mg), wollastonite for calcium (Ca), magnetite for iron (Fe), and jadeite for sodium (Na). Electron microprobe (EMP) was used to obtain quantitative chemical analysis of micron-sized crystallites and backscattered electron images, very helpful to find high-resolution spatial variations in the chemical composition of the samples. They were used in conjunction with spot probe analyses, either EDS or WDS methods. At least 16 to 30 points were analyzed for each specimen.

### 2.4. Scanning Electron Microscopy

A FEI Quanta 400 MK2 scanning electron microscope (SEM) was used for morphological investigations. Samples were fixed on a graphite stub with double-faced tape and investigated in high- and low-vacuum modes. In order to monitor that no artifacts associated with sputter coating were formed, morphological investigations were conducted on coated and uncoated samples. Samples were coated with a thin layer of graphite, as for the PTS, only for high-vacuum mode analysis. To investigate the internal structure of pathological mineralized deposits, some samples were fractured to allow analysis of freshly broken surfaces. X-ray microanalysis was carried out with an EDS Genesis XM2, manufactured by EDAX, and several measurements were taken for each sample. Four PTS were investigated by SEM as well, and X-ray maps of individual chemical elements were acquired at a beam energy of 15 kV using the K*α* lines of Ca, P, Na, Mg, and S, with a collection time of 2 hours.

### 2.5. Powder X-Ray Diffraction Analyses

Powder X-ray diffraction (PXRD) patterns were collected on a parallel X-ray beam Bruker AXS D8 FOCUS automated diffractometer, equipped with the parabolically shaped Göbel Mirror, using the Cu K*α*_1_ radiation (1.54051 Å) at 40 kV and 40 mA and operating in *θ*-2*θ* geometry. Data were collected in the 5–90° 2*θ* range with a step size of 0.02° and a counting time of 10 s at each step. Measurements were collected at room temperature in rotating 0.5 mm capillary tube. The samples were ground in an agate mortar into a fine powder. Two sets of diffractograms were collected: one on untreated powders and another on powders which have undergone deproteination treatment made to remove the organic component of the samples from the powders which significantly reduces the quality of the collected data. The deproteination treatment was performed through an enzymatic attack using trypsin in basic pH conditions. The cards from the International Center for Diffraction Data were used for phase identification.

The crystallite size as expression of mean diameter of the coherent-scattering domains (*D*) was determined with indirect measurements using diffraction peak broadening analysis. This was estimated using the Scherrer equation *D* = (*kλ*)/(*β*cos⁡*θ*) assuming (i) the line width/shape dominated only by particle-size effects, (ii) the Scherrer constant (*K*) as 0.9, and (iii) the peak broadening as Full Width Half Maximum (FWHM). The physical broadening of different diffraction lines, between ~25° and ~50° 2*θ*, was used after computer fit using the Gaussian line shape to determine the FWHM.

### 2.6. Statistical Analysis

Computations were performed with SPSS 19 (IBM, Armonk, NY, USA). Continuous variables are reported as mean ± standard deviation and categorical variables as *n* (%). Comparative analyses could not be performed due to the differences among groups.

## 3. Results

### 3.1. Polarized Light Microscopy Analyses

**Figure 2:**
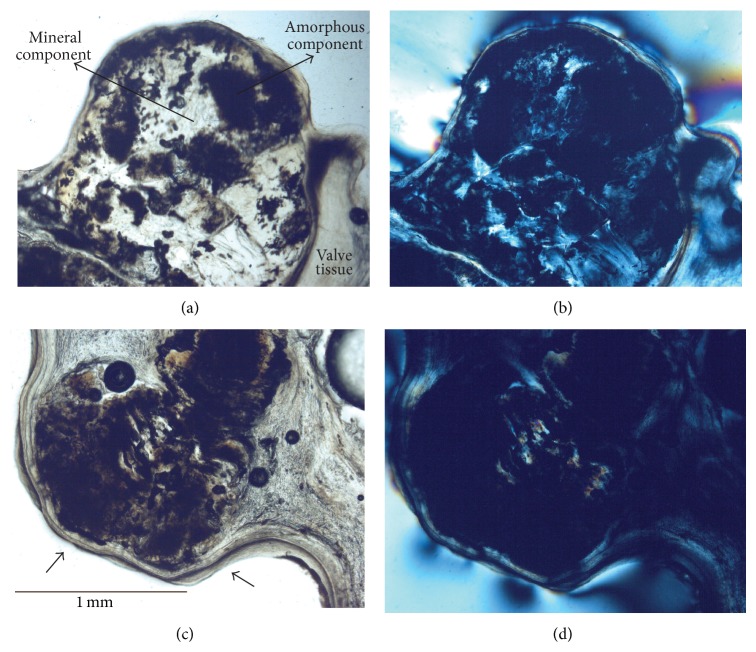
PLM analysis of valve nodules. Appearance of nodules of variable dimensions embedded in the valve tissue of a mineralized bicuspid aortic valve (Tv20ab) under transmitted PLM (a, c). (b, d) Under XPL the presence of extinguished zones is indicative of amorphous component. Zones having the typical first-order gray interference color are suggestive for apatite within the nodule. Contact between organic tissue and the epoxy resin was characterized by the presence of anomalous colors due to reaction between the two (b).

Under PLM, the ectopic deposit within heart valve tissue appeared as nodules of variable dimensions from 500 *μ*m to 2-3 mm completely embedded within valve tissue and made up of a mishmash of amorphous material and mineral (Figures 2(a) and 2(c)). Under cross polarized light (XPL) the latter had the typical first-order gray interference color of apatite (Figures 2(b) and 2(d)). Anomalous interference colors were sometimes seen at the sample interface, probably due to reaction between organic material and the epoxy resin used for polished thin section preparation.

### 3.2. Backscattered Electron Images and Scanning Electron Microscopy

**Figure 3:**
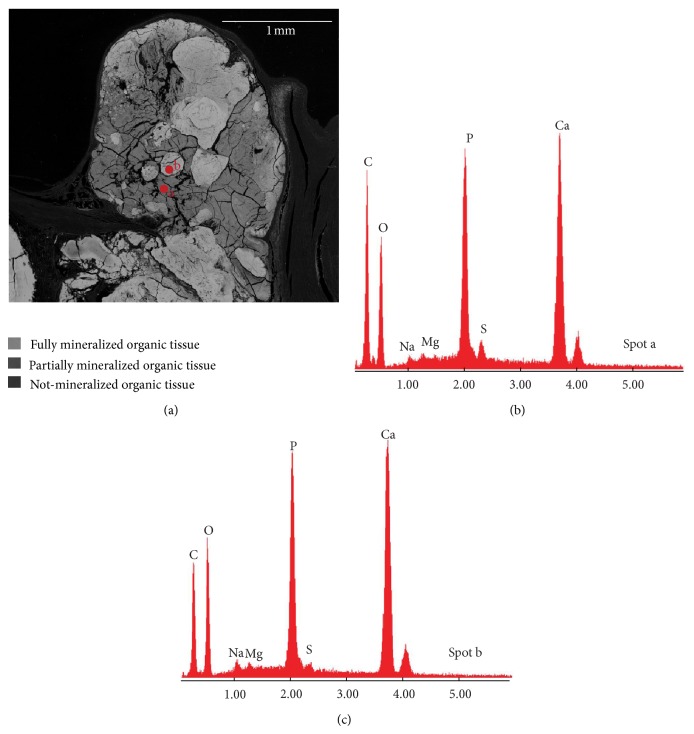
BSE image and EDS analyses of PTS (sample Tv20ab). (a) Millimeter nodule embedded in the heart valve tissue. The nodule appears to be formed by a massive mineral formation with a low brightness in which smaller nodules with higher brightness are visible. The EDS spectra of these zones with different brightness are given in panels (b) and (c); these show that Ca, P, and O are the main chemical elements that made up the heart valve mineralized tissues, confirming their calcium phosphate nature. Mg, Na, and S were also detected; the massive mineral formation characterized by a lower brightness (spot a) than micrometric nodules (spot b) had a greater content of carbon (C) and sulphur (S) and represents a partially mineralized tissue while the micrometric nodules represent fully mineralized areas. The darkest zones represent the organic matrix not mineralized (lowest mean atomic number), while the black zones represent voids in the embedding material.

**Figure 4:**
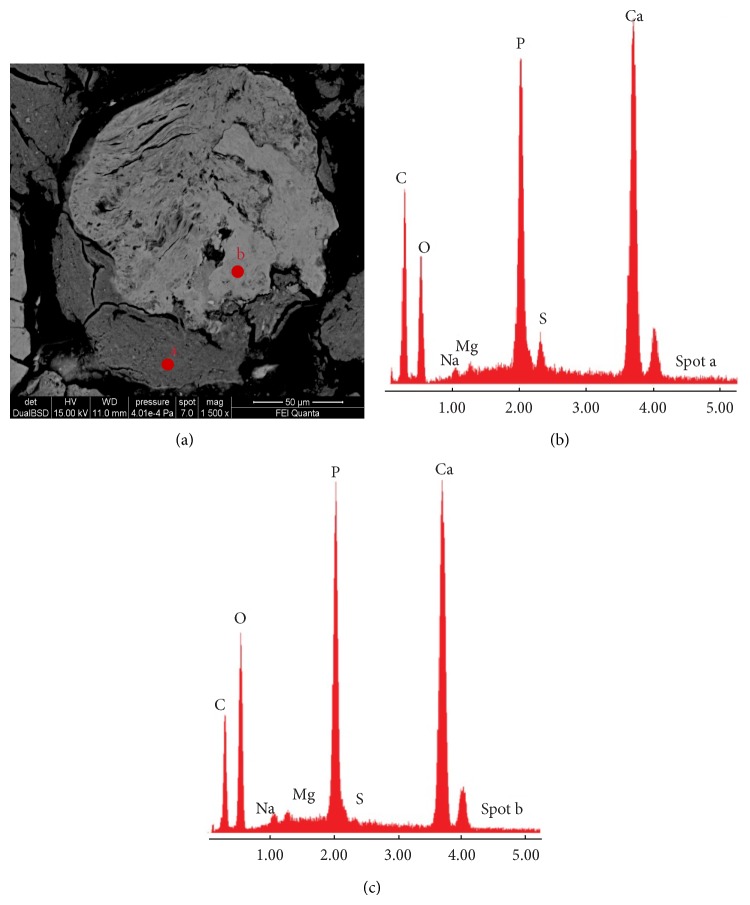
BSE-SEM analyses of PTS (sample Tv20ab). (a) High magnification image of a micrometric nodule formed by two different stages of mineralization. The lower zone of the nodule having lower brightness had greater content of C and S (spot a) (b) than the upper zone of the nodule (spot b) (c). This indicates a partially mineralized organic matrix for the lower zone of the nodule and a fully mineralized tissue for the upper zone.

Backscattered electron (BSE) images on PTS, obtained by either EMP or SEM-EDS analyses, confirmed the presence of millimeter nodular formations completely embedded in the heart valve tissue that instead appeared to be not mineralized. Within the millimeter nodules we observed areas with different brightness showing a grayscale range indicative of differences in mean atomic number (Figure 3(a)): a massive mineral formation with a lower brightness and micrometric nodules with higher brightness embedded within the massive mineral matrix. The presence of grayscale range allowed us to infer a nonuniform chemical composition of the mineralized tissue. Both micrometric nodules and the massive formation had the same chemical elemental composition, as indicated by energy dispersive spectrometry (EDS) spectra (Figures 3(b) and 3(c)), even if we noted a different amount of carbon (C) independently of the carbon coating and sulphur (S) between the light grey and the grey minerals in Figure 3(a). In particular the areas having lower brightness (lowest mean atomic number) were always associated with high content of C and S (Figure 4) indicating a greater amount of organic extracellular matrix (ECM).

**Figure 5:**
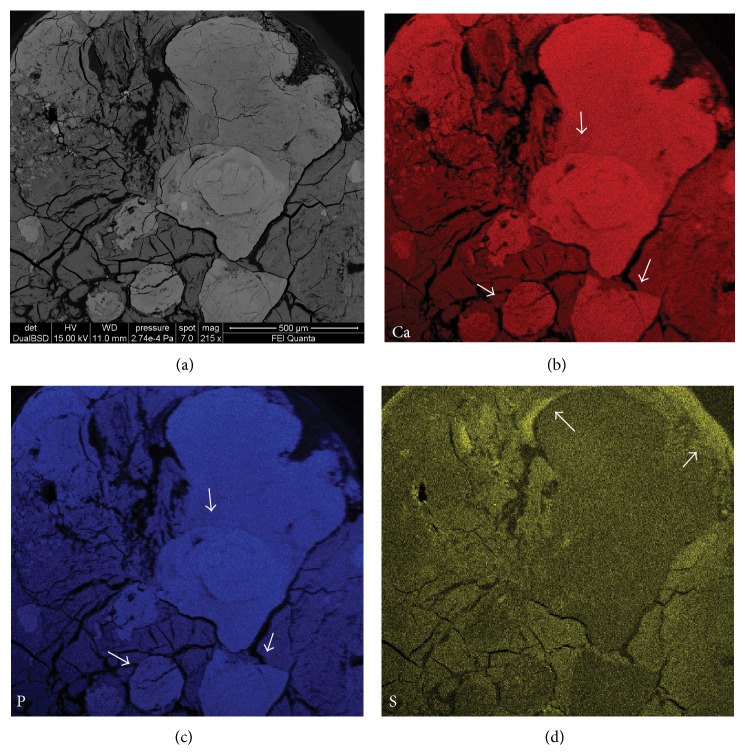
Elemental maps (sample Tv20ab). (a) BSE-SEM image of the area used to generate elemental maps. (b) Distribution of Ca. (c) Distribution of P. (d) Distribution of S. The arrows in panels (b) and (c) indicate completely mineralized areas characterized by very high concentrations of Ca and P. The arrows in panel (d) indicate high concentrations of S associated only with the extracellular organic matrix surrounding the pathological deposit.

Elemental mapping confirmed the presence of high concentrations of Ca and P within the brightest zones of Figure 3(a) and the presence of high concentration of S only in association with the ECM surrounding the pathological deposit. In contrast, the concentrations of Na and Mg were very low and probably below the detection limit of the instrument (Figure 5).

**Figure 6:**
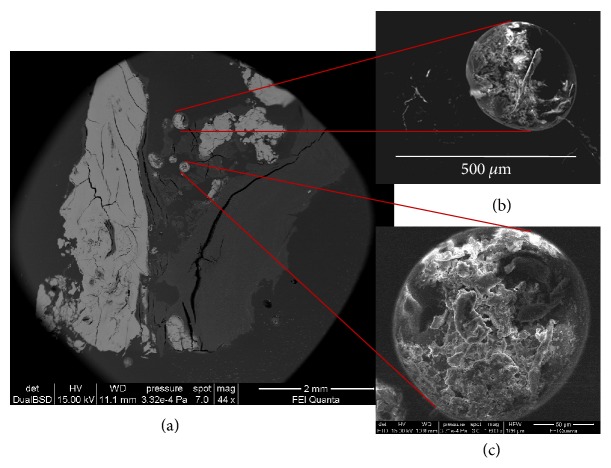
BSE-SEM analysis of circular cavities (sample Tv16 m). (a) Low magnification image of a PTS. The brightest areas represent fully mineralized zones. On the left, a massive and homogeneous deposit is visible, while in the center micrometric circular cavities are visible. (b, c) Magnified view of the small cavities indicated in panel (a); fragments of disorganized and mineralized collagen are visible within the cavities.

**Figure 7:**
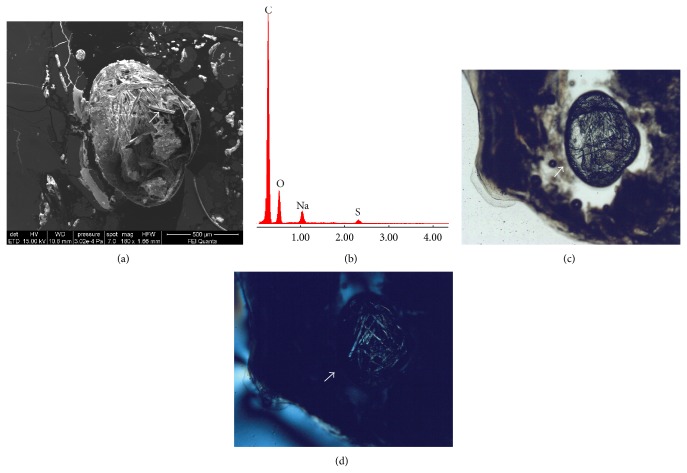
(a) SEM image (sample Tv12a) of a pocket filled only with proteins,* oxidized lipids*, and collagen fibers, formed by carbon (C), oxygen (O), sodium (Na), and sulphur (S) as indicated by the spectrum EDS in panel (b); the absence of P and Ca in the spectrum demonstrates the nonmineralized nature of the fibers. Images of the pocket under PLM (c) and XPL (d). The birefringent of the organic fibers within the pocket is clearly visible as well as their morphology very similar to amyloid fibrils.

Investigations on PTS brought to light also the presence of circular cavities (Figure 6) of variable size similar to vugs found within rocks, filled with fragments of disorganized and mineralized collagen (Figures 6(b) and 6(c)). Very large pockets filled with organic fibers formed by C, Na, and S without any trace of biomineralization, similar to amyloid-like fibrils [12, 13], were also observed (Figure 7).

**Figure 8:**
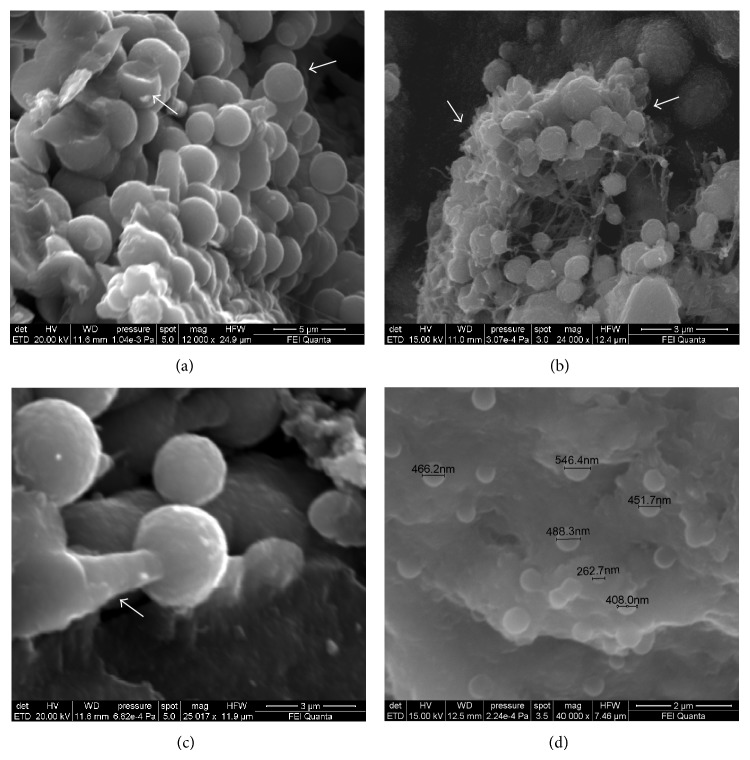
SEM analysis of pathological biomineralization morphology. (a) Agglomeration of variably sized (micrometer range) calcified nanoparticles, indicated by the arrows (12000x, mitral valve sample). (b) Agglomeration of spherical calcified nanoparticles in a framework of organic filaments (bicuspid aortic valve sample), formed by extracellular matrix proteins. (c) Magnified view (25000x) of individual spherical nanoparticle along a mineralized filament, indicated by the arrow (mitral valve sample). (d) Agglomeration of spherical calcified nanoparticles in the nanometer range at 40000x; the smallest measured nanoparticle was 262.7 nm in diameter (bicuspid aortic valve sample).

At high magnifications SEM analyses revealed the presence of coalescent spherical particles (Figure 8). The latter were variably sized and had a diameter from few micrometers up to nanometer range (Figure 8(d)). They were present in all the types of valves studied (bicuspid aortic valve, tricuspid aortic valve, and mitral valve), but in some samples they presented with a different degree of agglomeration.

High-resolution images also revealed a strict relationship between fine collagen fibers and spherical particles (Figure 8(b)). SEM analyses conducted on coated and uncoated samples confirmed the same results.

### 3.3. Electron Microprobe Analyses

**Figure 9:**
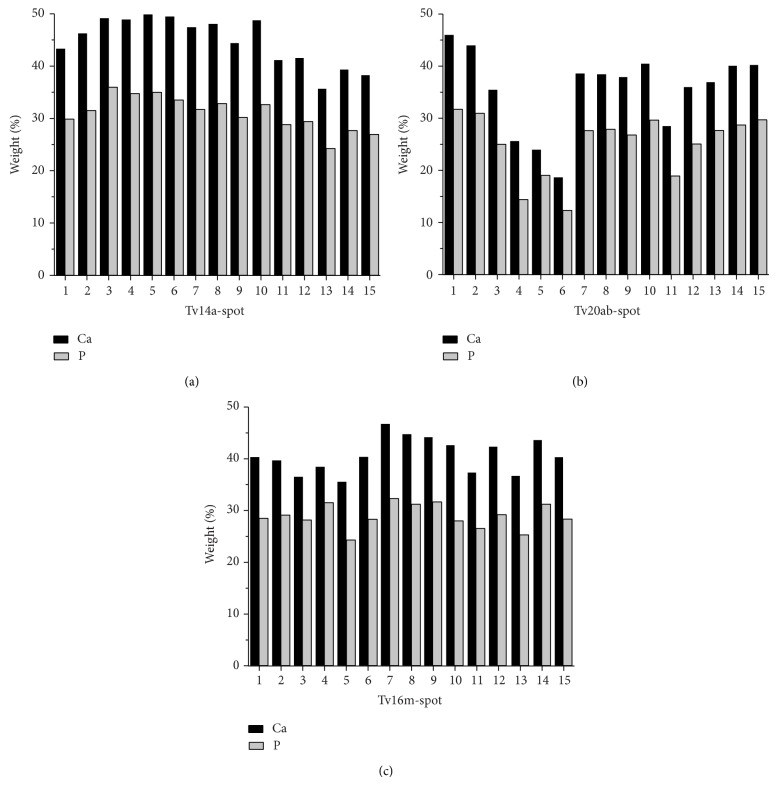
Chemical variability of Ca and P for three different calcified valves. The concentrations of Ca and P indicated in the histograms represent the concentrations determined in oxide-weight percent (CaO, P_2_O_5_) for fifteen punctual analyses (indicated as spot) taken within the same calcified deposit. Histograms showed that bicuspid aortic valves (b) were characterized by a lower content of Ca and P and by greater variations in Ca : P concentrations. In contrast, mitral (c) and tricuspid aortic valves (a) were characterized by a higher content of Ca and P and less variation in Ca and P concentration.

**Figure 10:**
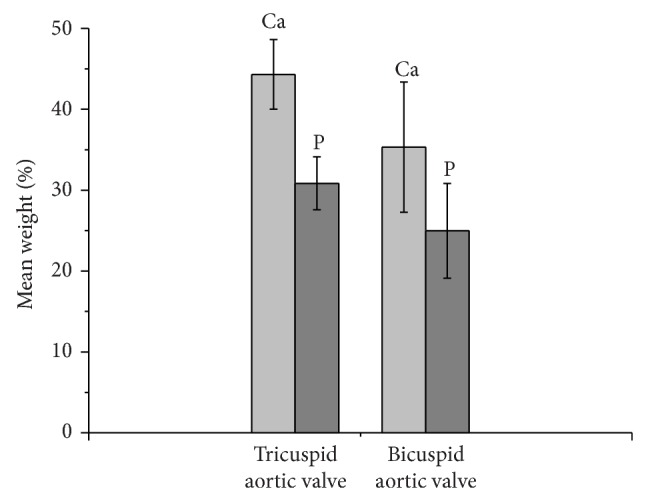
Comparison between two different types of calcified aortic valve. Histograms showed that the tricuspid aortic valve had a major content of Ca and P (expressed as mean weight %) compared to bicuspid aortic valve. The mean value was calculated on thirty spots taken within each valve. The vertical bars represent the standard deviation.

EMP data indicated that the chemical variability of each sample was great; Ca and P concentrations, as well as the atomic Ca : P ratio, changed significantly from point to point. In addition, there was substantial chemical variability also in the different types of heart valve studied. Calcified tricuspid aortic valves had the highest and most uniformly distributed Ca and P concentrations and a high Ca : P ratio; bicuspid aortic valves had the lowest Ca and P concentrations, with less uniform distribution of Ca and P within the calcified deposits; the mitral valve analyzed presented Ca and P concentrations similar to those in tricuspid aortic valves. The chemical variability of Ca and P is given in Figure 9, whereas their average content and the mean Ca : P ratio are given in Table 1. Moreover the comparison between the two different types of aortic calcified valves showed a greater content of Ca and P within the tricuspid aortic valve than bicuspid aortic valve (Figure 10).

Despite the variable concentrations of Ca and P in each sample, the atomic Ca : P ratio for each calcified valve was relatively uniform (Table 1). The calculated mean Ca : P values fell in the so-called “AB-type carbonated apatite” range, which corresponds to the presence of carbonate (CO_3_^2−^) in the apatite lattice [14]. Thus, the chemical variability of each sample seems to be strictly regulated by its carbonate content. This is not detectable by EMP, but we have well documented its presence with Raman and infrared spectroscopy published elsewhere [15].

### 3.4. Powder X-Ray Diffraction Analyses

**Figure 11:**
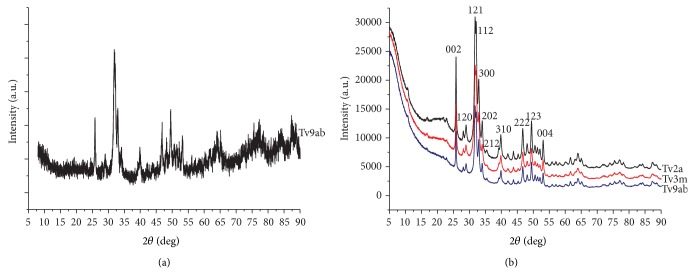
Experimental XRD patterns relative to three different types of mineralized human cardiac valves. (a) X-ray diffraction pattern of the untreated powders. (b) X-ray powder diffraction pattern of the biomineralization after the enzymatic attack. The main characteristic peaks match the hydroxylapatite pattern numbers 9–432 from the ICDS. The broadening of the peaks indicates a small crystal size within the nanometer range. a = aortic valve, ab = bicuspid aortic valve, and m = mitral valve.

X-ray diffraction patterns of the untreated powders showed a diffuse background due to organic component (Figure 11(a)); however the enzymatic attack cleaned successfully the powders from the organic matter and revealed how this is extended in the samples (Figure 11(b)). PXRD patterns confirmed the lattice structure to be consistent with that of standard hydroxylapatite, although the latter is significantly more crystalline. However the calcification appears to have a good crystallinity degree as indicated by the resolved diffraction peaks. All samples showed the same diffraction pattern and broadening of the diffraction peaks indicating very small crystallite size. According to Scherrer equation, all samples appear to be in the nanometer range with average crystallite domains size between 20 and 24 nm (see Supplementary Tables  1 and  2 in Supplementary Material available online at http://dx.doi.org/10.1155/2015/342984).

### 3.5. Feasibility

Several difficulties arise when studying the mineral component of calcified tissue and its organic matrix through purely mineralogical techniques. The main difficulties are linked to (1) the application of complementary experimental techniques to obtain reliable and interpretable results, (2) the very small amount of material, not allowing application of all experimental techniques, (3) the sample preparation procedures, and (4) the specific features of the pathological deposit under investigation; indeed calcium phosphate biominerals possess peculiar characteristics that make them very difficult to be analyzed. For example, these are soft and beam-sensitive materials and can be subjected to phase instability and artifacts formation. Therefore an accurate biomineralogical study such as this work is not easy.

To date application of EMP for studying standard geological materials such as inorganic crystals formed in abiotic systems is a routine analytical procedure; thus for calcium phosphate phases of geological environment recommended protocols and various operating conditions are indicated in the literature. Conversely, analytical protocols and sample preparation procedures for EMP analysis of specific biominerals are not still well defined. Therefore we made an accurate samples selection to evaluate the more suitable samples for each type of experimental analysis and then an evaluation of the optimal analytical conditions to obtain correct information. We used the same criterion for SEM analyses; moreover as the preparation of organic material for SEM investigations can introduce a variety of damage and artifacts, we made several morphological investigations, as described in the text, to monitor the formation of artifacts associated with the organic component of the samples.

## 4. Discussion

The first detailed report of aortic valve calcification was published by Moenckeburg in 1904, who proposed two different mechanisms to explain this phenomenon: (1) degeneration of the valve leaflet layers, originating near the sinuses of Valsalva and propagating toward the tips of the cusps, and (2) a sclerotic process of the aortic wall involving the cusps. Since then, a growing scientific body of evidence has been collected on the cellular and molecular mechanisms underlying calcification [16, 17]. Currently valve calcification is not considered a simple, passive, degenerative deposition of calcium phosphate complexes on the injured valve leaflet surface. Several mechanisms potentially promoting the cusps dystrophic calcification have been proposed, ranging from mechanical stress, inflammation, Ca/P homeostasis, focal energy dissipation, immune response, and metabolic changes to upregulation of osteogenic genotype. Progression of calcification seems to be mediated primarily by valve interstitial cells (VICs) [18, 19]. In particular, changes in VIC phenotype seem to have an important role in the pathogenesis of valve calcification [20, 21], as well as in the ECM maladaptations occurring with disease [22]. The calcifying osteoblast phenotype is considered the final common pathway of the heart valve calcification process [23], and the identification of osteoblast-specific transcripts supports the hypothesis that degenerative aortic valve stenosis can be considered the result of an active, multifactorial, highly regulated process similar to skeletal bone formation, although the regulation of these processes is fundamentally different [24].

One mechanism involved in the pathogenesis of valve calcification seems to be based on the activation of normally quiescent VICs in response to different environmental stimuli and on their differentiation into osteoblastic VICs [25]. Moreover, microRNAs are small noncoding RNAs implicated in several cardiovascular pathologies [26], including calcific aortic stenosis, where miR-26a, miR-195, and miR-30b were found reduced [27]; in particular, miR-30b attenuates bone morphogenetic protein 2-induced osteoblast differentiation by targeting Runx2, Smad1, and caspase-3 [28]. miR-30b has been proposed as a regulator of human aortic valvular calcification and apoptosis, regulating the osteoblastic VICs, but the exact link with the inorganic phase remains to be established. Recent findings demonstrated that cells from an extracardiac origin, such as circulating hematopoietic stem cells, may contribute to vascular and valvular calcification, by driving the differentiation processes. The presence of bone marrow-derived hematopoietic stem cells has been documented in adult valves, even if the function of these cells in valvular pathogenesis has not been defined [29]. Although much progress has been made in understanding the cellular mechanisms involved in the calcification process of human heart valves, it is not clear which signaling pathways are responsible for initiation and progression of this complex process. Despite the different biological mechanisms described in valve calcification, the inorganic phase has been less systematically analyzed. We aimed to apply the mineralogical point of view to heart valve calcification, to analyze the content of Ca and P and the physicochemical features of the biominerals included in the valve calcifications. Therefore, we report in detail the morphological and chemical features of the calcified deposits found on valve tissue. Chemically, these deposits are constituted by calcium phosphate with an apatite crystal structure very similar to hydroxylapatite. This is in agreement with the phase indicated in previous reports on human heart calcification [30–37]. Our elemental maps clearly show that the composition of the pathological phase is restricted to Ca and P, with a high content of CO_3_^2−^, very similar to the carbonate content of normal mineralized tissues, such as bone [15].

BSE images revealed the presence of different levels of calcification. We observed a heavily mineralized tissue, characterized by nodular formations, inside a mineral-deficient tissue in which the organic component was still dominant. The areas around the nodule formations represented this type of calcification. We consider these different levels of calcification as the expression of two different stages of the calcification process. In this sense, the mineral-deficient tissue appears to be in an initial stage of calcification and is indicative of an ongoing maturation process. We hypothesize that pathological mineral formation on valve tissue is a continuous process, similar to the multistep formation process of bone tissue [38–40]: as the organic part of the valve is lost, the mineral component increases. The brightest zones such as nodules were ascribed to the final bioapatite deposit, while the mineral-deficient tissue with minor brightness was ascribed to the initial stage of the process.

We also identified the presence of small cavities in the calcifications. High magnification images revealed the presence of disorganized tissue and in some cases of calcified and fragmented fibers, within these cavities. We consider these cavities of particular interest because they clearly show in three dimensions the degradation and abnormal remodeling of the ECM involved in the calcification process. We suggest that these localized zones, which are characterized by a disorganized matrix, represent a stage immediately preceding the formation of a heavily mineralized formation, such as bone-like nodule formations [29], and after the activation and transition of VIC phenotype.

Calcification appears to be morphologically organized in globular clusters of spherical particles. To date, the nature and mechanisms of nucleation and growth of spherical calcium phosphate particles in pathologic states are still unclear. It is still uncertain if these spherical particles are self-replicating life forms or if they derive from a physicochemical phenomenon without any relation to living organisms.* The so-called nanobacteria (or nanobacteria-like particles, nanobes, and calcifying nanoparticles)* have been considered* by mistake* for many years the pathogenic agents involved in the calcification processes, and this bacteriomorphic hypothesis was very diffuse [41–50]. However, it has been documented that* the nanoparticles observed in pathological calcification lack RNAs and metabolic activity* [50] but are associated with mineraloprotein* complexes as albumin and fetuin-A* [51, 52], also called calciprotein particles (CPPs) [52]. The estimated values obtained from powder X-ray diffraction analyses are in agreement with values of literature reported for similar “pathological” deposits [53] and are indicative of anisotropic crystallites longer in the* c*-direction than in the* a*- or* b*-direction, suggesting a preferential growth orientation and elongated shapes such as rod- or needle-like. In particular the measurements based on the peak linked to 002 reflection that is considered the most reliable peak for quantitative purposes, because of being well resolved and without overlapping with other peaks, are in agreement with the values estimated by Rusu et al. [54] for fully formed hydroxylapatite. We consider these spherical aggregates the morphological manifestation of calcifying nanoparticles of mineralo-protein and mineralo oxidized lipids that provide structural scaffolds for carbonate apatite crystals [55, 56] associated with a self-assembly process between organic and inorganic components [54]. In fact, the smallest spherical particle we measured was about 263 nm, whereas the mean size of crystallites calculated from PXRD was about 21 nm (see Supplementary Tables). Therefore, we propose that these biological aggregations are tiny bioapatite nanoparticles with crystals growing with the support of proteins as albumin, fetuin-A, and apolipoprotein A1, even if these types of nanoparticles simply adsorb any available protein from their surrounding environment [56]. At the same time, these spherical morphologies can be ascribed to extracellular vesicles- (EVs-) like particles [57, 58]. Their presence in calcified human heart valve supports the role of an active extracellular vesicular compartment in the formation of ectopic deposits within soft tissues.

However, complementary microanalytical techniques applied to harder minerals, such as focused ion beam, transmission electron microscopy, and laser ablation [59], are needed in order to obtain more information on these bioapatite nanoparticles. We plan to apply these techniques in further studies.

To the best of our knowledge, this is the first study to highlight the quantitative difference in Ca and P tissue concentrations between tricuspid and bicuspid aortic valves. The bicuspid aortic valve (BAV) is the most common congenital valve anomaly, with a prevalence of 2% in the general population and a male preponderance ratio of 2 : 1 [60]. The Ca and P content of tricuspid aortic valve (TAV) and mitral valve were similar, but the magnitude and the distribution of Ca and P were less in bicuspid valves (Figure 10). On the contrary, macroscopically, the congenital deformity of the BAV is associated with accelerated degeneration and heavier calcification than tricuspid, with a histoarchitecture of a rock-like mass of poorly mobile valve tissue. The Ca content of sinus, cusps, and arterial walls are similar in normal mammalian native semilunar valves [61] and the regional variation in normal semilunar valve tissue Ca concentrations is not predictive of later dystrophic calcification. The difference concentrations in calcified BAV are intriguing, as these valves are more subject to dystrophic calcification not only in the cusps, but also in the sinus and aortic wall. Impact of the asymmetrical hydraulic stress, inflammation, endothelial dysfunction, and neoangiogenesis probably affects more intensively the ECM when compared to tricuspid valves. It has been demonstrated that genetic factors affect the different behavior of aortic aneurisms in BAV and TAV cases [62], as in BAV there is a reduction in endothelial nitric oxide (NO) tissue endothelium levels, lower NO production, and increased release of MMP-2 and -9, determining endothelial dysfunction, activation of stretch and stress pathways, increased hypertension, increased apoptosis of smooth muscle cells, and weakening ECM. The molecular, cellular, and genetic differences between BAV and TAV affect also the Ca and P content, but further studies are needed to validate the biomineralogical difference of both types of aortic valve.

Understanding the features of pathological crystals is important if we are to obtain new insight into the mechanisms involved in valve calcification. This is fundamental for the development of therapies targeting the biomineralization process in order to improve the durability of surgically and transcatheter-implanted bioprosthetic valves. Invasive and costly surgical intervention and transcatheter implantation are today the only effective treatments of calcific valve stenosis.

## 5. Limitations

We are aware of the fact that a small sample size is the major limitation of this study. As described above this limitation is strictly linked to the methods of sample preparation for specific mineralogical techniques that require a very big amount of calcification, first of all to obtain accurate experimental results and to make reproducible analyses in order to verify their reliability when specific analytical protocols are missing. However, the large number of different and accurate analyses performed and the magnitude of the results are of relevance. Moreover, to our knowledge, this is the first report using pure mineralogical techniques applied to the study of soft bioapatite in humans. Studies comparing data from ultrastructural analyses, inflammatory markers, and biomineralogical data are ongoing and will be the object of future publications.

## Supplementary Material

Table 1: Average crystallite size is calculated for each diffraction peak.
Table 2: Unit cell parameters which are quite uniform among the different types of valve are presented.

## Figures and Tables

**Table 1:** Patients characteristics, experimental analyses performed (SEM-EDS, scanning electron microscopy with energy dispersive spectrometry; PXRD, powder X-ray diffraction; EMP, electron microprobe analysis; PLM, polarized light microscopy), and average content of calcium (Ca) and phosphorus (P) in different types of calcified valve. Ca and P contents are expressed as oxide-weight percent values and as atomic Ca : P ratio. Values are mean ± SD.

Characteristic	Overall *N* = 36	Tricuspid aortic valve *N* = 29	Bicuspid aortic valve *N* = 3	Mitral valve *N* = 4
Age, y	72.4 ± 10	74.5 ± 7.8	55 ± 15	69 ± 9
Males	25 (69.4%)	20 (69%)	3 (100%)	2 (50%)
SEM-EDS	20 (55.5%)	13 (45%)	3 (100%)	4 (100%)
PXRD	27 (75%)	23 (79%)	2 (66%)	2 (50%)
EMP	5 (14%)	2 (7%)	2 (66%)	1 (25%)
PLM	5 (14%)	2 (7%)	2 (66%)	1 (25%)
CaO	40 ± 5.7	44.3 ± 4.3	35.3 ± 8.2	40.7 ± 3.4
P_2_O_5_	28.1 ± 4.2	30.85 ± 3.3	25 ± 6	28.85 ± 2.3
Ca : P	1.8 ± 0.08	1.82 ± 0.05	1.8 ± 0.105	1.78 ± 0.09

## Abbreviations

BAV: Bicuspid aortic valve
BSE: Backscattered electron microscopy
ECM: Extracellular matrix
EDS: Energy dispersive spectrometer/spectrometry
EMP: Electron microprobe
PLM: Polarized light microscope/microscopy
PTS: Polished thin sections
PXRD: Powder X-ray diffraction
TAV: Tricuspid aortic valve
SE: Secondary electron
SEM: Scanning electron microscope/microscopy
VICs: Valve interstitial cells
WDS: Wavelength dispersive spectrometer/spectrometry
XPL: Cross polarized light.
