# Supplementary material for: Morphological and Chemical Study of Pathological Deposits in Human Aortic and Mitral Valve Stenosis: A Biomineralogical Contribution
Source: Patholog Res Int. 2015 Jan 19;2015:342984. doi: 10.1155/2015/342984 (PMC4313546; doi:10.1155/2015/342984)
Supplement: Supplementary file 1 — Table 1: Average crystallite size is calculated for each diffraction peak. Table 2: Unit cell parameters which are quite uniform among the different types of valve are presented. [file 342984.f1.docx]

**Supplementary Table 1.** Average crystallite size estimated for the pathological deposits of the human valve tissues **by the Sherrer equation.**

| **Sample** | **D_002_**  (nm) | **D_120_**  (nm) | **D_121_** (nm) | **D_300_** (nm) | **D_212_** (nm) | **D_310_**  (nm) | **D_113_**  (nm) | **D_222_** (nm) | **D_213_** (nm) | **D_004_** (nm) | **Average crystallite size** (nm) |
| --- | --- | --- | --- | --- | --- | --- | --- | --- | --- | --- | --- |
|  | **2θ**  25.81 | **2θ**  28.95 | **2θ**  31.8 | **2θ**  32.9 | **2θ**  39.23 | **2θ**  39.86 | **2θ**  43.80 | **2θ**  46.72 | **2θ**  49.45 | **2θ**  53.27 |  |
| **Tv2a**  FWHM (°) | 37 0.22 | 20 0.40 | 15 0.55 | 16 0.52 | 21 0.40 | 16 0.51 | 28 0.30 | 20 0.43 | 23 0.38 | 27 0.33 | 22 (7) |
| **Tv3m**  FWHM (°) | 26 0.26 | 26 0.31 | 10 0.85 | 16 0.51 | 21 0.41 | 15 0.55 | 23 0.37 | 18 0.47 | 21 0.42 | 26 0.34 | 20 (5) |
| **Tv6a**  FWHM (°) | 37 0.22 | 23 0.34 | 16 0.51 | 17 0.49 | 22 0.38 | 17 0.50 | 28 0.31 | 21 0.41 | 24 0.37 | 29 0.31 | 23 (6) |
| **Tv9ab**  FWHM (°) | 37 0.22 | 23 0.35 | 17 0.48 | 17 0.48 | 21 0.39 | 19 0.45 | 30 0.28 | 22 0.39 | 24 0.36 | 28 0.31 | 24 (6) |
| **Tv12a**  FWHM (°) | 33 0.25 | 26 0.32 | 16 0.52 | 16 0.50 | 22 0.38 | 16 0.51 | 25 0.34 | 22 0.40 | 23 0.38 | 26 0.34 | 22 (5) |
| **Tv14a**  FWHM (°) | 34 0.24 | 25 0.32 | 14 0.59 | 15 0.55 | 22 0.39 | 16 0.52 | 23 0.37 | 21 0.41 | 23 0.38 | 27 0.33 | 22 (6) |
| **Tv15m**  FWHM (°) | 33 0.25 | 21 0.39 | 10 0.81 | 15 0.55 | 21 0.40 | 15 0.57 | 28 0.31 | 19 0.46 | 21 0.42 | 27 0.33 | 21 (7) |
| **Tv18ab**  FWHM (°) | 35 0.23 | 22 0.38 | 16 0.52 | 16 0.51 | 22 0.39 | 17 0.50 | 30 0.29 | 21 0.41 | 23 0.38 | 29 0.31 | 23 (6) |

*a = aortic valve, †m = mitral valve, ‡ab = bicuspid aortic valve, **D = mean diameter of the coherent-scattering domains derived by the Sherrer equation, FWHM= the Full Width Half Maximum for the diffraction peak under consideration; 2θ= the Bragg angle.**

The unit cell parameters revealed little variations from geological hydroxylapatite and from bioapatite of mineralized tissues. The *a* parameter contraction and the *c* elongation detected can be associated to the presence of CO_3_^2-^ group in the apatite lattice as we have previously documented [S. Mangialardo, V. Cottignoli, E. Cavarretta, L. Salvador, P. Postorino, A. Maras, “Pathological biominerals: Raman and Infrared studies of bioapatite deposits in human heart valves,” *Applied Spectroscopy*, vol. 66, pp. 1121-1127, 2012].

**Supplementary Table 2.** Cell parameters calculated for pathologic bioapatite (Space Group: *P6_3_/m*)

| **Sample** | **Sex, Age** | ***a*(Å)** | ***c*(Å)** |
| --- | --- | --- | --- |
| Tv2a* | M, 75 | 9.4172(5) | 6.8954(4) |
| Tv3m† | F, 81 | 9.4161(8) | 6.8948(6) |
| Tv6a | M, 75 | 9.4153(8) | 6.8952(7) |
| Tv9ab‡ | M, 71 | 9.4165(7) | 6.8957(6) |
| Tv12a | M, 68 | 9.4165(8) | 6.8951(7) |
| Tv14a | M, 69 | 9.4161(9) | 6.8958(7) |
| Tv15m | M, 64 | 9.4161(9) | 6.8961(7) |
| Tv18ab | M, 41 | 9.4158(5) | 6.8958(4) |

*a = aortic valve, †m = mitral valve, ‡ab = bicuspid aortic valve
